# Using intervention mapping (IM) to develop a self-management programme for employees with a chronic disease in the Netherlands

**DOI:** 10.1186/1471-2458-10-353

**Published:** 2010-06-21

**Authors:** Sarah I Detaille, Joost WJ van der Gulden, Josephine A Engels, Yvonne F Heerkens, Frank JH van Dijk

**Affiliations:** 1 Seneca, Expertise Centre for Sports, Work and Health, HAN University of Applied Sciences, Nijmegen, The Netherlands; 2 Department of Primary and Community Health, Radboud University, Nijmegen Medical Centre, The Netherlands; 3 Faculty of Health and Social Studies, HAN University of Applied Sciences, Nijmegen, The Netherlands; 4 Faculty of Health and Social Studies, HAN University of Applied Sciences, The Netherlands; 5 Coronel Institute of Occupational Health, Academic Medical Center, University of Amsterdam, The Netherlands

## Abstract

**Background:**

Employees with a chronic disease often encounter problems at work because of their chronic disease. The current paper describes the development of a self-management programme based on the Chronic Disease Self-Management programme (CDSMP) of Stanford University to help employees with a chronic somatic disease cope with these problems at work. The objective of this article is to present the systematic development and content of this programme.

**Methods:**

The method of intervention mapping (Bartholomew 2006) was used to tailor the original CDSMP for employees with a chronic somatic disease. This paper describes the process of adjusting the CDSMP for this target group. A needs assessment has been carried out by a literature review and qualitative focus groups with employees with a chronic disease and involved health professionals. On the basis of the needs assessment, the relevant determinants of self-management behaviour at work have been identified for the target population and the objectives of the training have been formulated. Furthermore, techniques have been chosen to influence self-management and the determinants of behaviour and a programme plan has been developed.

**Results:**

The intervention was designed to address general personal factors such as lifestyle, disease-related factors (for example coping with the disease) and work-related personal factors (such as self-efficacy at work). The course consists of six sessions of each two and a half hour and intents to increase the self management and empowerment of employees with a chronic somatic disease.

**Conclusion:**

Intervention mapping has been found to be a useful tool for tailoring in a systematic way the original CDSMP for employees with a chronic somatic disease. It might be valuable to use IM for the development or adjusting of interventions in occupational health care.

## Background

In 2007, 39 percent of the U.S. working age population had at least one chronic disease such as diabetes, asthma or depression [1]. Prognostic studies predict an increase in the next twenty years of the incidence of chronic diseases like asthma, chronic obstructive pulmonary diseases (COPD), diabetes and rheumatoid arthritis (RA) in the working population [2,3].

Many people with chronic diseases are able to lead productive lives if supported to do so. However, a chronic disease, such as RA or COPD, has a multidimensional impact on peoples' lives, which can result in limitations in performing activities of daily life and at work, and therefore in job loss or permanent work disability [4-8]. In the Netherlands only one third of the people between the ages of 16 to 64 with a chronic disease have a paid job in comparison to two thirds of the general population [4]. Despite improvements in facilities and medical care, thirty percent of the employees who have a chronic disease have problems at the workplace related to the disease [9,10].

In traditional occupational health interventions, the client had a rather passive role. In the past decade, occupational health interventions have also focussed on empowerment and health promotion among employees [11,12]. In the Netherlands, recent disability pension legislation has made employees themselves more responsible for job retention [13].

In a systematic review Verbeek et al [14] proposed to classify the outcomes of occupational health intervention programmes into three categories: 1) exposure change, 2) skills and behaviour change, and 3) disease and disability change. Most existing occupational health intervention programmes are based on skills and behaviour change and have focused on empowerment at the workplace, like acquiring psychological support, communication skills, training in requesting work accommodations and on feelings of self-confidence or self-efficacy in dealing with work-related problems [11,15,16].

Different studies based on the patient's perspective which have been used in the needs assessment (see method and results section) provide information that employees with a chronic disease need to acquire empowerment skills to cope with the problems encountered at work because of their chronic disease. There is some evidence that occupational health interventions for employees with a chronic disease based on the empowerment perspective are effective [17].

There are several programmes available for the empowerment of people with a chronic disease. One of the most frequently used programmes is the Chronic Disease Self-Management programme of Stanford University (CDSMP) developed by Lorig et al. in 2006 [18]. The CDSMP is an example of a lay-led health education programme aimed at helping participants develop a range of skills and confidence to deal more effectively with their chronic conditions [19]. A Cochrane review on the effectiveness of such self-management programmes by lay leaders [20] shows that these programmes can lead to short-term improvements in patients' confidence to manage their condition and perceptions of their own health. There were also significant improvements in cognitive symptom management of pain, disability, fatigue and depression [20]. The CDSMP has been shown to improve self-efficacy, self-management behaviour and health status, while reducing hospitalization and emergency visits [21-23]. The original CDSMP focuses on personal factors like lifestyle and disease-related factors like coping with symptoms of the disease. As this programme does not include work-related factors such as self-management behaviour at work, it doesn't fit entirely the needs of employees with a chronic disease.

In this study the method of intervention mapping (IM) [24] was used to adjust the original CDSMP for employees with a chronic somatic disease. The method of IM has been successfully applied earlier to develop a workplace intervention for sick listed employees with stress-related mental problems and musculoskeletal disorders [25,26] and a worksite physical intervention [27]. The focus of this paper is a detailed overview of how IM was used to develop a self-management programme for employees with a chronic somatic disease.

## Methods

### Intervention mapping

**Figure 1:**
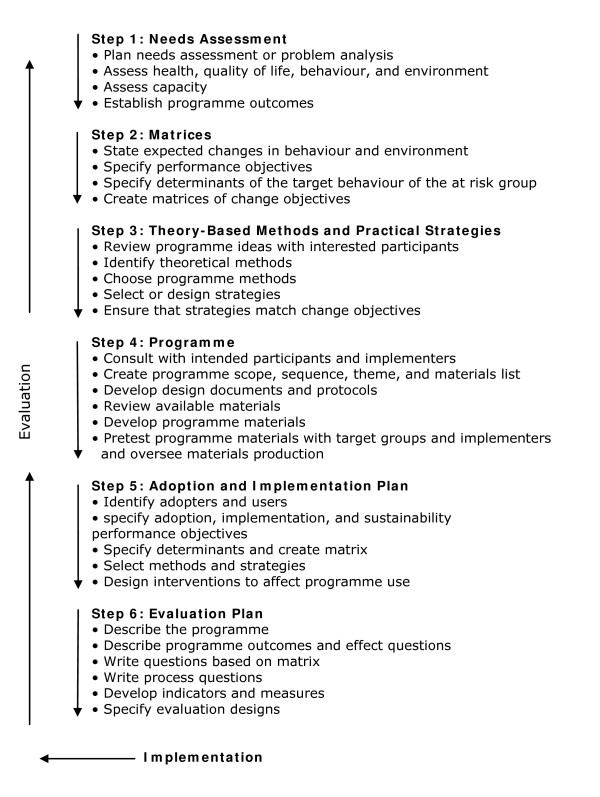
**Intervention mapping protocol**.

IM is a stepwise approach for theory and evidence based development and implementation of interventions. It comprises six steps, each leading to a product that guides the next step (see Figure 1) [24]. The present paper focuses mainly on the results of step one to four (creation of intervention) and also on the boundary conditions in order to carry out the intervention in the context of the evaluation study (step five and six).

#### Step one: Needs assessment, literature review, and focus groups

In *step one *a needs assessment is conducted starting with assessing the health problem. The health problem concerned has to be serious and must be related to behaviour. In order to detect modifiable factors, individual and environmental determinants of the risk behaviour are investigated (table 1). These investigations involve the application of behavioural determinants theories such as the theory of planned behaviour (1985) (which includes elements of Social cognitive theory of Bandura, 1977) and the protection motivation theory of Rogers (1975) [24,28].

**Table 1:** Identified important determinants of self-management behaviour at work.

Personal determinants	Socio-cultural determinants at work	Environmental determinants
Attitude towards asking help at work if needed	Attitude of supervisor	Type of job
Attitude towards coping with symptoms of chronic disease	Attitude of colleagues	Autonomy at work
Self efficacy for asking for help	Modelling behaviour of colleagues	Work tasks/content
Self efficacy for coping with symptoms	Relation with supervisor/colleagues	Social relations at work
Self efficacy for planning work	Culture of company	Working conditions
Awareness of risks of unhealthy lifestyle	Social relations at work	Facilities at work

In the present study, firstly focus group interviews have been carried out with employees with RA, diabetes and hearing loss (n = 69) to explore the prerequisites for employees with a chronic disease to function at work [29-31]. Secondly, the same question was explored through concept mapping with different health professionals (occupational health physicians, occupational health and specialist nurses, family doctors and specialists n = 54) [29-31]. Thirdly a systematic literature review was conducted to determine which prognostic factors are related to work disability in employees with a chronic somatic disease [32].

The needs assessment provided the information needed to be able to screen the content of the original CDSMP and compare this with the content needed for our target population.

#### Step two: Identification of outcomes, performance objectives and change objectives

*Step two *provides the foundation for the intervention by specifying who and what will change as a result of the intervention.

**Figure 2:**
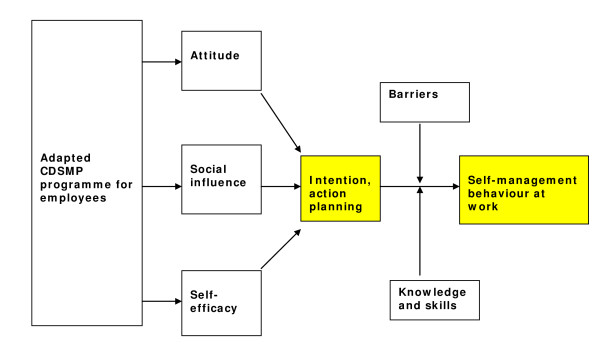
**Determinants of behaviour model**.  Model representing how the adapted CDSMP programme can influence determinants of self-management behaviour at work, including the impact of barriers, knowledge and skills.

In order to analyze the determinants of self-management behaviour at work in this study the theory of planned behaviour (TPB) [33-35] was applied. The TPB postulates that intention, the most proximal determinant of behaviour, is determined by three independent constructs: attitude, social influence and perceived behavioural control (self-efficacy) (Figure 2) [33-35]. In this step we defined the overall desired outcome of the adjusted intervention. The product of step two is a set of matrices of change objectives that combine performance objectives for personal and environmental determinants. Change objectives are the most immediate targets of an intervention [24].

#### Step three: Selecting methods and practical strategies

In *step three *theory-informed methods and practical strategies to change the behaviour of individuals are gathered. Change theories and theory-based change strategies (table 2) are then used to assess the changeability of the determinants. Examples of behavioural change theories are the goal-setting theory and social cognitive theory [24].

**Table 2:** Methods for determinants translated in intervention.

Determinant	Method	Strategies
Attitude	Belief selection	Through awareness exercises, based on brainstorm sessions and discussions, participants learn to identify current beliefs on having a chronic disease, their lifestyle and the problems encountered at work
	Decisional balance	Awareness exercises and brainstorm sessions also take place to identify possible solutions

Self-efficacy	Goal setting	Participants formulate at the beginning of the course a long term and a short term goal Through weekly action plans participants can work on their self-efficacy and their long term goal
	Modelling	Participants are (positively) influenced by the achievements of other participants

Social influence and support	Modelling Guided practice Social comparison	Through weekly action plans participants can mobilize social network and social support at work

Knowledge	Information about the positive effects of a healthy lifestyle	Information in textbook Presentations Group sessions
	Information about determinants of work disability for employees with a chronic disease	Information in textbook Presentations Group sessions

Risk perception (skills for identifying high-risk situations)	Confrontation with risk of unhealthy behaviour (lifestyle and behaviour at the workplace)	Through awareness exercises, based on brainstorm sessions and discussions, participants learn to identify risks behaviours
	Modelling	Course leader shares examples
	Skill training, with guided practice and feedback	

Skills for developing coping, problem solving and negotiation	Modelling	Through awareness exercises based on brainstorm sessions and discussions participants learn to identify barriers and how to handle them
	Skill training with guided practice and feedback	Skills practice in role plays with feedback

In this step of our study we have reviewed the literature on the CDSMP to be able to identify the effectiveness of methods and strategies used in the original CDSMP programme. For each determinant of behaviour appropriate methods were identified from the literature [24,34]. The methods and strategies of the original CDSMP were compared to the performance objectives for the intervention formulated in step two.

#### Step four: Creating an organised programme plan

The products in *step four *include a description of the scope and sequence of the components of the intervention.

For our study we compared the performance objectives of the original CDSMP to the performance objectives formulated for the adapted version of the CDSMP. The objectives on lifestyle behaviour and coping with the disease were already included in the original CDSMP, as well as the aspect "communicating with health professionals". The theme "problems encountered at the workplace because of a chronic disease" was not included in the original CDSMP. Therefore we developed two additional sessions on what is needed to be able to work with a chronic disease and how to communicate with ones supervisor, colleagues and occupational health professionals about the problems encountered at work. In this step a plan was created for the adjusted intervention.

#### Step five: Creation of an adoption and implementation plan

The product of *step five *is a plan for accomplishing programme adoption and implementation.

In our study, several actions were taken to prepare the use of the programme in an evaluation study. Promotion material and a plan for the recruitment of participants for the training and the control group for the evaluation study were developed. It was ensured that facilitators received the correct training and instructions in order to carry out the training.

#### Step six: Creating an evaluation plan

In the final step of intervention mapping an evaluation plan

In this step we have developed a plan for the quantitative and qualitative evaluation of our evaluation study. The results of the evaluation study will be presented and discussed elsewhere when available.

## Results

### Step one: Needs assessment

#### Focus groups

Three focus groups with employees with RA, diabetes and hearing loss (total n = 69) were held to explore the prerequisites for employees with a chronic disease to function at work [28-30]. The same question was explored through concept mapping with different health professionals (occupational health physicians, occupational health and specialist nurses, family doctors and specialists; n = 54). For the employees with RA, the support of management was the most important, followed by self-acceptance, self-efficacy, and professional advice on how to cope at work. For employees with diabetes mellitus, self-acceptance, self-care, and support from management, colleagues and health professionals were rated to be the most important. For the employees with hearing loss, to have knowledge of hearing aids was the most important, followed by communication strategies, the ability to cope with the disease, to be assertive and the support of occupational physicians [29]. According to the health professionals [30,31], employees with a chronic disease need self-management skills to manage their disease (for example to accept their illness, to communicate about it with others and to feel confident enough to work). A supportive family and work-environment was also perceived as an enabling condition for work. Furthermore support from health professionals and a balance between workload and capacities were considered to be important. The findings of the focus groups also suggest that occupational health interventions should pay attention to personal factors, disease related factors and psychosocial factors at the workplace.

#### Literature review

Different risk factors for work (dis)ability have been identified in the literature for employees with a chronic somatic disease like RA, Ischemic heart disease, asthma or COPD: a physically demanding job, precision work, lack of control over the pace and activities of work, lack of support by supervisors, managers and colleagues and complexity of work. Disease related factors such as pain, fatigue and poor functional status are important factors for leaving the workplace [32,36-39]. Informing colleagues about having the disease seems to be positively associated with continuing to work as well as adjusting job demands and behavioural coping at work [40]. Systematic reviews on prognostic factors for work-(dis)ability support the consideration that many personal, disease-related and work-related factors are common for certain chronic physical diseases [32,41]. The findings of the literature review suggest that occupational health interventions should pay attention to personal factors, disease related factors and psychosocial factors at the workplace.

Factors such as the competence to ask for support, to adjust working demands, to accept the disease and to cope with the disease at work can probably be influenced by a self-management programme [11,42]. Based on the findings of the focus groups and literature review we decided to screen the content of the original CDSMP and compare the content to the needs of our target population. The original CDSMP focuses on personal factors like lifestyle, self-efficacy and skills to communicate about the disease. The original CDSMP also focuses on disease-related factors like coping with symptoms of the disease. The results of the needs assessment give insight that the original CDSMP already covers many aspects which are important for employees with a chronic disease. The only aspect which is not incorporated in the original CDSMP is work-related factors such as self-management behaviour at work. We decided therefore to adjust the original CDSMP to fit the needs of employees with a chronic disease. The next step was to identify and formulate personal, socio-cultural and environmental determinants for self-management behaviour at work (Table 1). The personal determinants are possibly directly influenced by the course and can possibly indirectly affect the socio-cultural and environmental determinants at work.

### Step two: Matrices of change objectives

Based on the needs assessment, the overall behavioural outcome was defined as "Self-management behaviour at work".

The aim of the CDSMP for employees with a chronic somatic disease is to obtain self-management behaviour at work. *Self-management behaviour at work *has been operationalized as follows:

1), To be able to ask help from colleagues and supervisor when needed (ask for facilities at work, ask for change in job demands).

2), To be able to cope with symptoms as pain, fatigue, breathing problems and emotional ups and downs at work and carry out a healthy lifestyle.

3), To be able to re-organize work according to disease (to plan work according to disease, to take pauses when needed and to say no when needed).

Furthermore the main determinants of behaviour change according to the theory of planned behaviour [33]; (1) the attitude, (2) social influence and (3) perceived behavioural control to behaviour (Figure 2) have been operationalized as follows:

1. *Attitude*: A person's attitude consists of the perceived cognitive and emotional advantages and disadvantages of the behaviour, including beliefs that a specific type of behaviour can be completed. How positive is the person about the capability to ask support from colleagues and supervisor at work, to cope with symptoms as pain, fatigue, breathing problems and emotional ups and downs, to carry out a healthy lifestyle and to reorganize work according to the disease.

2. *Social influence*: (perception of) social support at work and acquiring social support at work. Social influences consist of the perception of others carrying out this type of behaviour (social modelling), the norms that people have with respect to these behaviours (social norms) and the support that they perceive from others in carrying out a particular type of behaviour.

3. *Self-efficacy*: how confident is the person on his ability to ask for support when needed at work, to cope with symptoms at work, to carry out a healthy lifestyle and to reorganize work according to the disease. Self-efficacy refers to a person's perception of his capability to carry out the type of behaviour.

The intervention is aimed to influence all three determinants of behaviour but specially the self-efficacy at work. According to the theory of planned behaviour, behaviour is best predicted by the intention of the person to perform that behaviour [24]. Recent findings support that action planning is a better predictor of behaviour than the intention [43]. Interventions are proven to be more effective if they focus on improving participants' action planning activity, their self-efficacy and self regulatory capabilities rather than focusing on intention-enhancing risk perceptions [44,45].

### Step three: Theory-based methods and practical strategies

There is systematic evidence available on effective methods to stimulate self-management behaviour at work. A systematic review on the effectiveness of empowerment interventions at the workplace [16] showed that most existing interventions have the objective:

• to increase knowledge (about the disorder and its consequences, legal rights and work accommodations)

• to gain a clear understanding of work-related problems or work barriers

• to increase feelings of control (general control or perceived self-efficacy in the process to request work accommodations)

• to develop skills (coping skills and social competences)

• to increase activities aimed at work accommodations

The objectives of existing empowerment interventions at the workplace focus primarily on acquiring skills and behaviour change. The CDSMP programme for employees focuses on skills and behaviour change by improving participants' action planning activity, self-efficacy and self regulatory capabilities as well as influencing their intention and risk perceptions. In table 2 the different techniques used in the course to influence the determinants of behaviour are shown.

The techniques for behavioural change used in the self-management programme to influence the determinants are: consciousness raising (belief selection, decisional balance), risk perception, positive reframing, self-re-evaluation, enhancing self-efficacy and social support, skill mastery, reinterpretation of symptoms, goal setting, social comparison, modelling, and persuasion of positive outcomes [24,46,47].

The *attitude *of the participants is influenced by awareness exercises to raise their consciousness on situations at the workplace which are difficult to deal with a chronic disease. Participants are encouraged to formulate possible solutions. Self-management behaviour is also influenced by the attitude and actions of the other participants.

The *social support *at work is influenced by encouraging employees to talk about the course and action plans with colleagues and supervisor.

The *self-efficacy *is influenced by social comparison [48,49] through success stories of other participants. Through goal setting (action plans) the participants can focus on working on their self-efficacy, based on the level of the participant. Goal setting leads to better performance because people with explicit goals exert themselves to a greater extent and persevere in their tasks [50,51]. A goal should be behaviourally SMART formulated (specific, measurable, attainable, realistic and timely) and should be stated in terms of behaviour (ask for help at work) instead of health outcomes, e.g. oriented on more social support from colleagues [47,52]. Participants formulate weekly a goal with regard to self-management behaviour, for example, to exercise, to practice time management at work or to take pauses at work, which they intend to accomplish during the following week. After formulating the plan, the participant has to state how confident he is that he will execute the action plan. If the level of confidence is below 7 (on a 1-10 scale), the participant is coached in re-formulating his action plan by the course leaders until a higher level of confidence is achieved [47]. During the next session, the participants report whether or not they have accomplished their action plan, and to give an account if any possible problems that might have arisen are solved. This feedback is an integral part of skills mastery.

### Step four: programme

In step four, we created a modified plan for the programme taking into account the budget and resources for the programme materials. The course consists of six sessions of each two and a half hour, this conform the programme plan of the original CDSMP. An overview of the adapted CDSMP for employees is shown in table 3. For this target group, two extra sessions have been developed based on the model of work load and work capacity [53] by using the methods of the original CDSMP (e.g. consciousness raising, risk perception, positive reframing, skill mastery, goal setting). Furthermore the original CDSMP topics were enriched with new topics on the work situation of employees with a chronic disease.

**Table 3:** Content of the self-management programme for employees with a chronic somatic disease.

*Timing*	*Lesson*	*Topics*
*Week 1*	*Introduction* *Importance of physical exercise*	- Overview of the course
		- Objectives of the course
		- Objectives of the participants
		- Inventory of problems encountered at work by the chronic disease
		- Introduction to cope with symptoms by using guided imagery
		- The importance of physical exercise for people with a chronic disease
		- Introduction to making action plans

*Week 2*	*Coping with pain, fatigue and stress at work*	- Symptoms that interfere with the ability to work
		- Situations causing stress, pain or fatigue (at work)
		- Solutions to deal with stress, pain or fatigue (at work)
		- Breathing exercises
		- Introduction to cognitive symptom management

*Week 3*	*Importance of healthy nutrition/Problems encountered at work*	- Introduction to healthy nutrition
		- The importance of healthy nutrition for people with a chronic disease
		- Introduction to working with a chronic disease
		- Introduction to the model of work load and work capacity
		- Solutions at the workplace

*Week 4*	*Communication techniques at the workplace*	- Communication techniques
		- How to communicate with supervisor and colleagues about the problems encountered at work
		- How to communicate with supervisor and colleagues about possible solutions at work
		- How to communicate with family and friends about the problems and possible solutions to combine work and home

*Week 5*	*Working together with occupational health professionals*	- Working together with occupational health professionals and HRM advisors at work

*Week 6*	*Plans for the future*	- What has been accomplished the past six weeks?
		- What have we learned in the course?
		- Formulating long-term plans

The programme plan has been produced conform the boundary limits of the original CDSMP. The original programme is intended for (lay)-trainers who have completed the Master trainers programme at Stanford University. The original CDSMP design is a high feasible low-cost programme which can practically be implemented in every setting. The only boundary limits for the programme are: two trainers (one must be a master trainer at Stanford University and the other trainer must have received a leaders training by the master trainer), inset time approximately 5 hours per session (training plus preparation time) and an accessible accommodation (room) for minimal 15 participants and facilities like access to beverages, toilet access and an elevator.

### Step five: Adoption and implementation plan

The results of step five were a well defined set of inclusion criteria for the participants, a plan for the recruitment of participants for the training in the context of the evaluation study, a plan to train the facilitators and a Dutch manual for the participants and the facilitators [54].

The inclusion criteria to select participants for the course were: employees with a diagnosed chronic physical disease, with a paid job at the moment of the course, who encounter problems at work because of their disease and who were motivated to follow the course. The exclusion criteria were: Employees with predominant psychiatric conditions, more than three months totally absent from work and fully work-disabled.

Participants for the course were recruited through the departments of Human Resource Management from companies, general practitioners and occupational health services in the region of Arnhem and Nijmegen in the Netherlands. An information letter and leaflet of the course were sent to 82 companies, 88 general practitioners and 10 occupational health services in both municipalities. Also several advertisements have been placed in regional newspapers. Participants were requested to contact the researcher (SD) by telephone or email for more information or to apply for the programme. Before being admitted to the programme participants were screened on the inclusion criteria by telephone. After registration the participants received a written confirmation, the informed consent form, the questionnaire and information about the procedures. All participants who have been admitted to the programme by telephone were randomized to either the control group or the intervention group. The control group consisted of care as usual and the intervention group consisted of care as usual plus the self-management programme. Both groups were followed for eight months.

Participants in the control group who were followed for eight months and had returned all the questionnaires in the control group and still wanted to apply for the self-management programme were allowed to follow the programme. The data of these participants has been included in the analysis of the control group and excluded from the analysis of the intervention group in the evaluation study.

We modified the course handbook for the participants and translated the manual in Dutch under the title "Werken met een chronische aandoening" (Working with a chronic disease) [54]. We also produced a manual for the facilitors including step by step instructions on how to implement the intervention. The course must be facilitated by two moderators. One of them is to be trained at the University of Stanford to be a master trainer of the CDSMP.

### Step six: Evaluation Plan

The result of step six was an evaluation plan for the evaluation study. The study design and operationalization of the evaluation study have been approved by the Medical Ethics Committee of the Radboud University Nijmegen and are registered in the Dutch Trial Register as (NTR 1737).

The effect-evaluation consists of a randomized controlled trial (n = 104) with eight months follow-up and a qualitative evaluation among the participants of two training groups (n = 15). In this study, we wanted to include at least 35 patients in the RCT in each group in order to be able to detect a statistically significant difference on the outcome SF-12 and coping with symptoms (Stanford questionnaire coping with symptoms). This sample size was based on a intervention study, in which 35 patients in each group were needed in order to achieve an effect-size of 0.8 on the SF-12 with a power of 80% and an alpha at <5% (two tailed) [55,56]. Assuming a drop-out rate of 20% during the trial a total of 104 participants have to be included in the randomization process.

Our primary analysis was by intention to treat and participants were at random selected for the control group or the intervention group. After selection they were informed about the intervention and control conditions. The control group received care as usual and the intervention group consisted of care as usual plus the self-management programme. A questionnaire has been developed including primary and secondary outcome measures. The primary outcome measures of the effect evaluation are self-efficacy at work, the intention to communicate with supervisor and occupational physician, work pleasure (VBBA) [57] and work productivity (WAI) [58], coping with symptoms like pain and fatigue (Stanford questionnaire coping with symptoms) and Quality of life and general health (SF-12) [59]. For the purpose of measuring self-efficacy at work, a self-efficacy at work instrument has been developed. The content of this questionnaire has been developed with the information obtained through the focus groups with professionals and patients.

The qualitative evaluation study consists of semi-structured interviews with fifteen participants. The participants were interviewed two times, at the beginning and after the course. Secondly, all brainstorm topics generated during the course related to the problems encountered at work and the solutions will be analyzed using content analysis.

The results of the RCT and the qualitative evaluation will be presented, when available, in separate articles.

## Discussion

We aimed to describe the development and content of a theory- and practise-based prevention programme for employees with a chronic somatic disease. Intervention mapping (IM) was used to develop an intervention based on the Chronic Disease Self-Management programme (CDSMP). Following each IM step carefully, made it possible to adapt the existing CDSMP programme to the needs of employees with a chronic disease. A systematic approach for the development is needed in order to build a theory-based intervention that fits the needs of a specific population [24,33,34]. At this stage it is not possible to draw conclusions about the effectiveness of the programme and the best context to diffuse the programme.

To our knowledge this is the first study, which has applied IM for the development of an occupational health intervention for employees with a chronic somatic disease. IM has been proven to be a helpful tool to screen existing interventions like the original CDSMP of Stanford University and to tailor the intervention for a specific population. Although IM provides a useful checklist to take the right steps in developing an intervention, it does include the risk to be a time-consuming process according to the IM textbook, especially for the development of totally new interventions. In our study, we experienced that IM is a useful checklist to relatively quickly tailor an existing intervention that has already been proven to be effective in helping participants to cope with their disease in general. The adapted intervention plan was completed in three months (analyses of step 1 to step 4). IM can be used in occupational health to screen and adjust existing interventions for different target groups [25,26].

A possible strength of the IM approach is the use of theories and methods for behaviour change like for instance the theory of planned behaviour which includes elements of the social-cognitive theory and goal-setting theory; these have been chosen in our study to influence the determinants of behaviour [24]. Recent insights in occupational health support the use of such conceptual models when developing evidence-based occupational health interventions [14]. Conceptual models are needed to construct an intervention tailored for the needs of a specific population and avoid trial and error procedures which may cost even more time and resources in the long-run.

Another possible strength of the programme is the generic character of the training. The information from the needs-assessment supports the idea that the determinants for work-ability are the same for different chronic somatic diseases. The generic character of the programme contributes to the feasibility of the programme in different contextual settings.

A difficulty might be that the theoretical knowledge and the experience with techniques that can be applied to influence the determinants of behaviour are insufficiently available in the field of occupational health care to apply IM. Another point of concern is the time available in health care settings to construct interventions according to the IM protocol. Wolfers et al. 2007 propose therefore a flexible and more pragmatic use of IM while still guaranteeing the quality of a systematic approach [60].

A possible weakness of our study is that the vision of employers has not been taken into account in the development of the intervention. The intervention has been constructed based on evidence found in the literature and on the vision of employees and health professionals. Probably, employers can mention relevant points not addressed yet during the training. Another point of discussion is whether a self-management programme for the employee is sufficient to facilitate the work-ability of employees with a chronic disease or whether the physical and social working environment should also be the object of an intervention.

The validity of the programme in different contextual settings like a company or occupational health service or in different countries is not studied yet. However, the content of the programme is to our knowledge transferable to different contextual settings under the condition that the target population and objectives of the contextual setting are the same as the target population and objectives of the present programme. Nevertheless we recommend screening the programme according to the IM checklist (figure 1) before using the programme for a different target population.

Another point of concern is that the training is not tailored for participants according to a stage of behavioural change and one specific behavioural goal. The course has been build to suit the needs of people in different stages of behavioural change and participants work on different behaviour goals. Probably this can cause a problem when evaluating the effects of the training on a specific behaviour.

To date, IM has been mainly used as a tool for the planning and development of health promotion interventions to suit the needs of a specific target population. Recently, promising results were shown for the use of IM in occupational health care research [25,26]. This study shows that IM can also be used to adjust existing generic programmes for specific working populations.

An article describing the methods and results of the RCT and recommendations for the diffusion of the programme will be submitted in the near future. An article on the qualitative evaluation is already submitted.

## Conclusion

In this study the original Chronic Disease Self-management programme has been tailored for employees with a chronic disease by adding new elements. The method of Intervention Mapping provided the tools needed to do this systematically. IM seems to be a proper method for the systematic development (or adjustment) of interventions in (occupational) health care.

## Competing interests

The authors declare that they have no competing interests.

## Authors' contributions

SD carried out the study, participated in the design of the study, performed the analyses and drafted the manuscript. YH and JE participated in the design of the study and helped to draft the manuscript. JG and FD participated in the design of the study, discussed interpretation of results and helped draft the manuscript. All authors read and approved the final manuscript.

## Pre-publication history

The pre-publication history for this paper can be accessed here:

http://www.biomedcentral.com/1471-2458/10/353/prepub
